# Macrophages are targets of retinoic acid signaling during the wound-healing process after thulium laser resection of the prostate

**DOI:** 10.18632/oncotarget.18238

**Published:** 2017-05-26

**Authors:** Dian-Jun Yu, Xing-Jie Wang, Yun-Feng Shi, Chen-Yi Jiang, Rui-Zhe Zhao, Yi-Ping Zhu, Li Chen, Yuan-Qing Yang, Xiao-Wen Sun, Shu-Jie Xia

**Affiliations:** ^1^ Department of Urology, Shanghai General Hospital of Nanjing Medical University, Shanghai 200080, China; ^2^ Department of Urology, Shanghai General Hospital, Shanghai Jiao Tong University School of Medicine, Shanghai 200080, China; ^3^ Institute of Urology, Shanghai Jiao Tong University, Shanghai 200080, China; ^4^ Department of Urology, Ningbo Medical Center Lihuili Eastern Hospital, Ningbo 315048, China; ^5^ Department of Urology, Wujin Hospital Affiliated Jiang Su University, Changzhou 213302, China

**Keywords:** benign prostatic hyperplasia, wound healing, retinoic acid, RARβ, macrophage

## Abstract

**BACKGROUND:**

The wound-healing process is very important for reducing complications after thulium laser resection of the prostate (TmLRP). The retinoic acid (RA) signaling pathway has been well studied in the wound-healing process of the skin and other organs. The goals of this study were to identify the role of RA signaling in the repair of the prostate after TmLRP and to investigate the molecular mechanism of this process.

**RESULTS:**

Retinoic acid receptors (RARs) were present in the prostate, and their expression was increased after TmLRP. RARβ was expressed in the macrophages and may be related to the role of stromal cells in the wound-healing process. In vitro, RA enhanced the function of anti-inflammatory macrophages and promoted stromal cell activation and angiogenesis. *Arg1* was also increased via RARβ after treatment with RA.

**MATERIALS AND METHODS:**

The expression of RARs was analyzed *in vivo* by immunohistochemistry (IHC), real time qPCR, and western blot analysis. THP-1 cells were co-treated with or without RA and stimulating factor and then assessed by ELISA and qPCR. The supernatants from these cells were cultured with stromal cells and vascular endothelial cells, and the effects on these cells were analyzed.

**CONCLUSIONS:**

We found that RA signaling was involved in the wound-healing process of the prostate after TmLRP. RA treated macrophages activated stromal cells and promoted angiogenesis. RARβ was the key isoform in this process.

## INTRODUCTION

Benign prostatic hyperplasia (BPH) is a common cause of lower urinary tract symptoms (LUTS) in men over 40 years of age. Approximately 20% of all BPH patients will eventually require surgery to relieve their symptoms [1]. Transurethral resection of the prostate (TURP) is advocated as the gold standard to treat symptomatic BPH according to contemporary guidelines [2]; however, TURP carries the risk of significant complications, such as bleeding and TUR syndrome (TURs). Thulium laser resection of the prostate (TmLRP) is a safe procedure that produces minimal tissue damage, bleeding, and TURs [3]. However, 23.1% of patients have symptoms of dysuria and urgency and even complications such as bladder contracture and urethral stricture after TmLRP [3]. The wound-healing process facilitates surgical wound closure and reduces these complications. This process includes cleaning necrotic tissue, activating stromal cells, angiogenesis, and re-epithelialization. An understanding of the wound-healing process after TmLRP and further determination of a method to accelerate the process are of great value. Previous studies have identified the importance of re-epithelialization in this process [1]; in the current study, we investigated the stromal cell and macrophage interactions in wound healing.

Retinoic acids (RAs) are a group of natural and synthetic derivatives of vitamin A that are known to play a crucial role in cell proliferation, differentiation, and maintenance of the homeostasis of the prostate [4, 5]. RAs have various stereoisomers, including all-trans-RA (ATRA), 9-cis-RA (alitretinoin), and 13-cis-RA (isotretinoin). RAs are transcriptional activators that bind to intracellular retinoic acid receptors (RARs) and regulate gene expression via RA responsive elements. Three RAR isoforms have been described: RARα, -β, and -γ [6]. RAs induce the formation of prostatic buds from the urogenital sinus (UGS) to initiate prostate growth and regulate the process via binding to RARs to increase sonic hedgehog (Shh) expression and by decreasing bone morphogenetic protein 4 (Bmp4) [7]. Changes in RA concentrations, as well as in RARs expression, have been described during BPH and prostate cancer, suggesting that this pathway plays an important role in the prostate [4]. The RA signaling pathway has been widely studied in the healing of cutaneous wounds [8], acute kidney injury, and spinal cord contusion injury [8–10]. In their study, topical application of RA significantly improved wound healing of cutaneous wounds by promoting M2 macrophages polarization [8]. The prostate wound healing process was thought to be similar to skin wound repair in our previous studies [1]. We explored the role of the pathway in wound healing of the prostate after TmLRP.

## RESULTS

### RARs were present in the canine prostate

**Figure 1:**
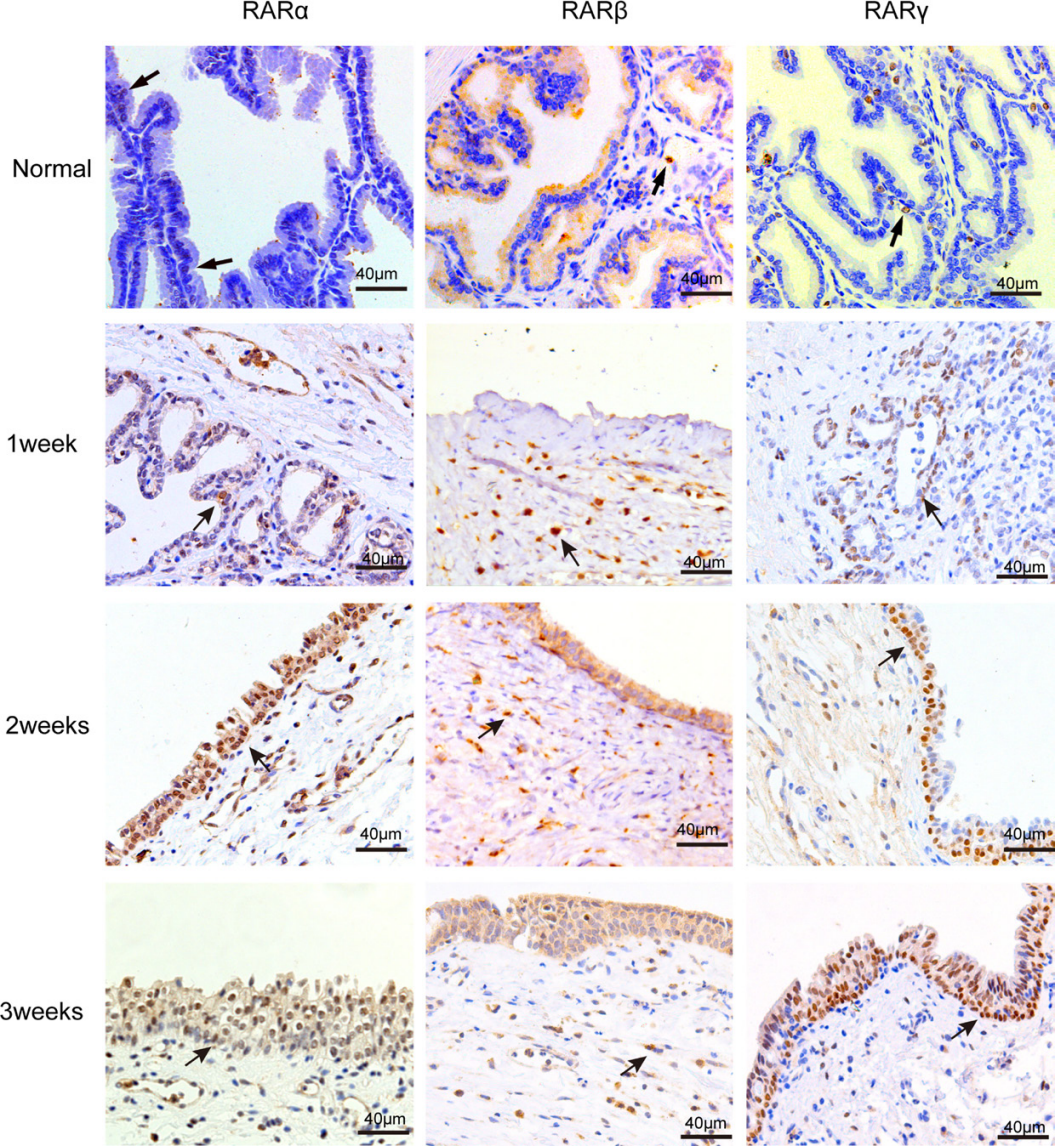
IHC staining of RARs on normal canine prostate and the wound area after TmLRP RARα-positive and RARγ-positive cells were mainly present in the epithelial cells of prostate glands. RARβ-positive cells were found in the stroma prostate. And the number of positive cells increased after TmLRP.

To address the question of whether RARs are expressed in the canine prostate, immunohistochemistry (IHC) was performed on unoperated canine tissue. We found that a small number of RARα-positive cells were present in the epithelial cells in normal canine prostate glands (Figure 1). Very few RARβ-positive cells were found in the stroma of the normal canine prostate. RARγ-positive cells were expressed mainly in the epithelial cell layer and nominally expressed in the stroma.

### The expression of RARs was increased after TmLRP

**Figure 2:**
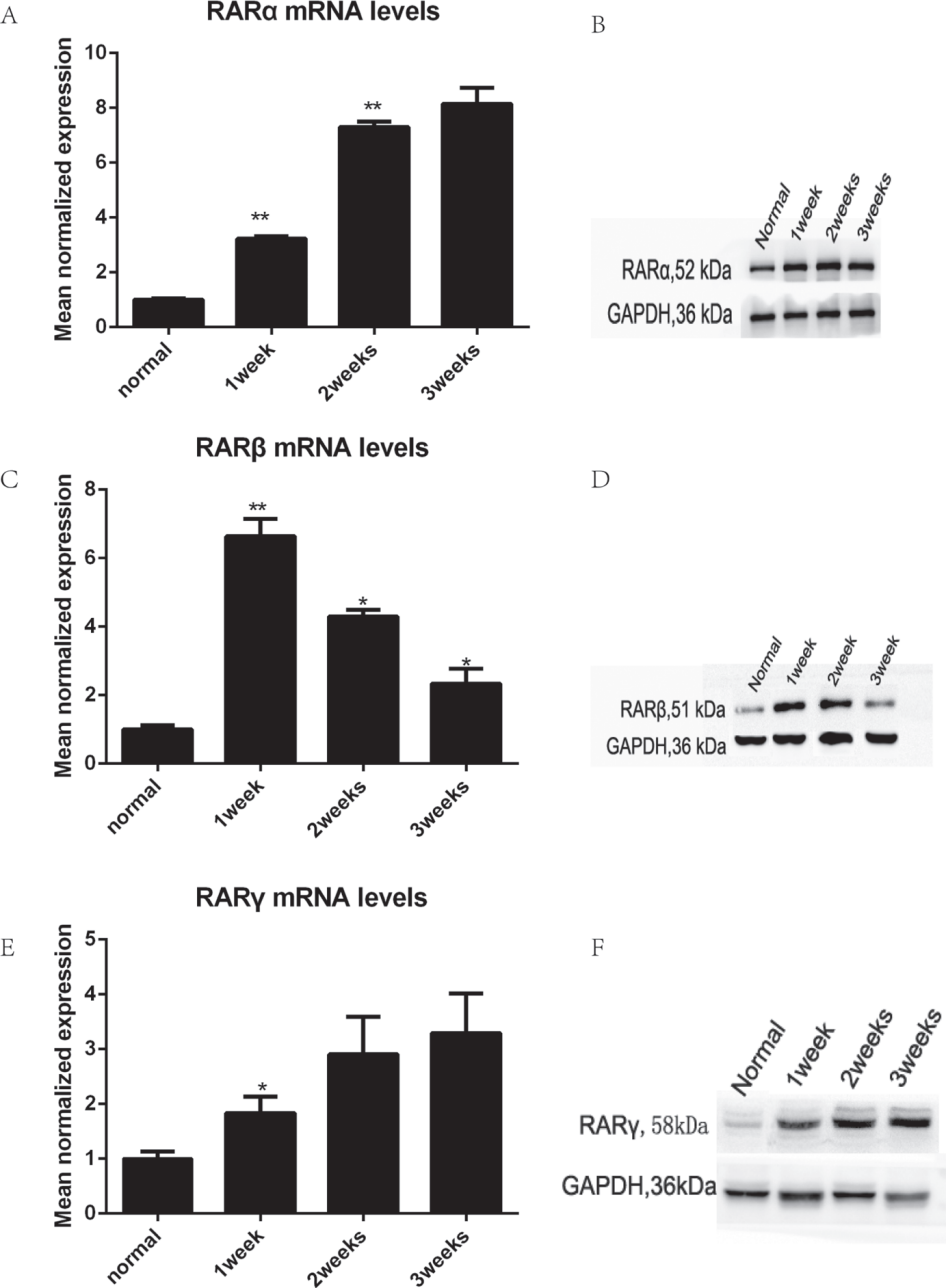
The expression of RARs was increased after TmLRP analyzed with western blotting and real-time qPCR (**A**, **B**, **E**, **F**) RARα and RARγ steadily increased after TmLRP. (**C**, **D**) RARβ levels increased markedly during the first week and then decreased.

Protein and mRNA extracted from wound specimens were analyzed with western blotting and real-time qPCR. The results showed that all types of RARs were present in the canine prostatic wound specimens (Figure 2). TmLRP caused an increase in the total amount of RARs during the first week after surgery. RARα and RARγ steadily increased during the second and third weeks after TmLRP (Figure 2A and 2B, 2E and 2F). However, RARβ levels increased markedly during the first week and then decreased during the second week. The expression was approximately equivalent to the preoperative level during the third week (Figure 2C and 2D).

### Cellular distribution of RARs in the prostate wound after TmLRP

**Figure 3:**
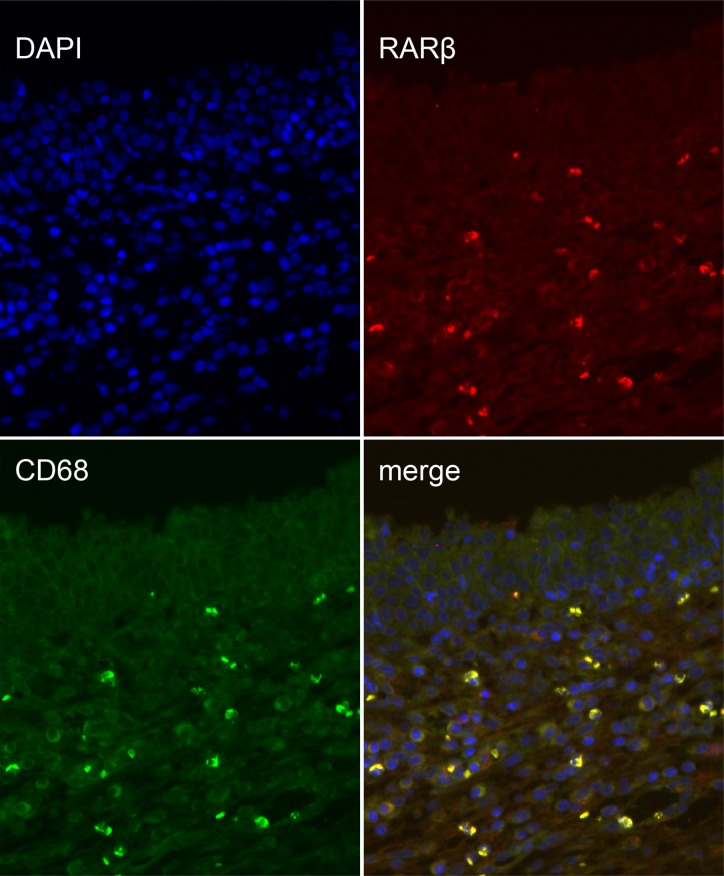
Double immunofluorescence of CD68 and RARβ in the prostatic wound CD68 was used as a macrophage marker (green, CD68; red, RARβ).

**Figure 4:**
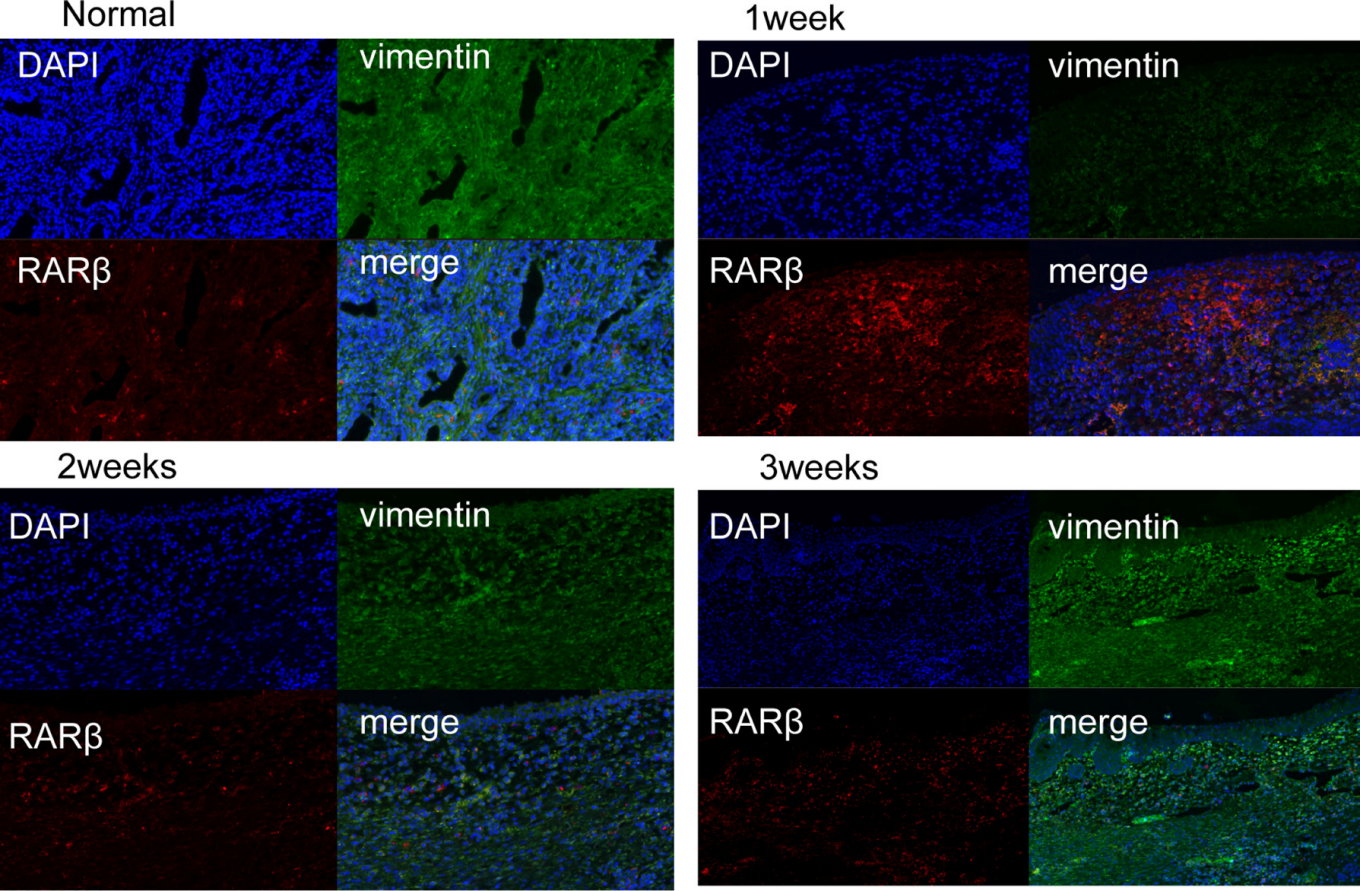
Double immunofluorescence of vimentin and RARβ in the prostatic wound vimentin was used as a stroma marker (green, vimentin; red, RARβ). A significantly increased number of RARβ-positive cells was observed during the first week after TmLRP, which then gradually declined, and few cells were observed during the third week. However, the presence of vimentin indicated that stromal cells were gradually expanding in number.

To analyze the cell types that express RARs in the wound-healing area, we investigated their distribution via IHC analysis of unoperated canine tissue and at 1, 2, and 3 weeks after TmLRP. In the wound-healing area, IHC staining for RARα was present in epithelial and stromal cells. RARγ occurred mainly in the basal layer of epithelial cells, with slight expression in the stromal cells. Expression of RARβ was seen in the macrophages in the prostatic stroma. To further confirm this finding, immunofluorescence results were examined (Figure 3). A significantly increased number of RARβ-positive cells was observed during the first week after TmLRP, which then gradually declined, and few cells were observed during the third week. However, the presence of vimentin indicated that stromal cells were gradually expanding in number (Figure 4). The results implied RARβ-positive cells may promote the process of wound healing in stroma. Further *in vitro* studies were performed to evaluate this finding.

### RA enhances anti-inflammatory macrophage activation *in vitro*

**Figure 5:**
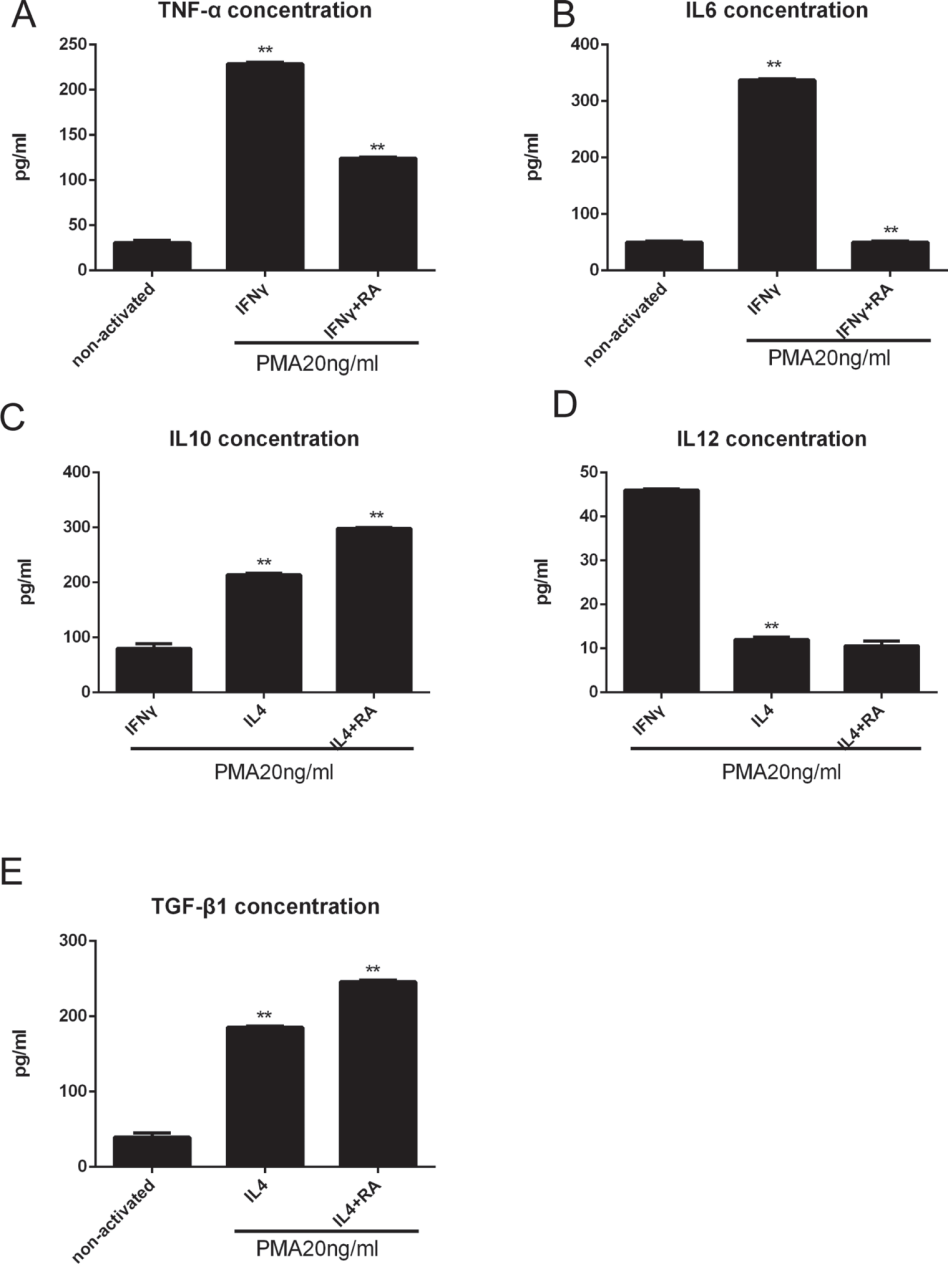
RA enhances anti-inflammatory macrophage activation *in vitro* analyzed by ELISA (**A**, **B**) THP-1 cells were treated with PMA (20 ng/ml) for 24 h, and then IFN (100 U/ml) with or without all-trans-RA (RA100nM) was added for 24 h, the TNF-α and IL6 levels were decreased. (**C**–**E**) THP-1 cells were treated with PMA (20 ng/ml) for 24 h and then treated with IL-4 (100 U/ ml) for 24 h with or without RA (100 nM), the TGF-β and IL10 levels were increased, but IL12 level was descent. *p < 0.05 and **p < 0.01 as compared to the control group.

To investigate the potential effects of RA signaling on the wound-healing microenvironment, we focused our interest on stromal cells because the re-epithelialization of epithelial cells is highly dependent on their surrounding cells and tissue. To better clarify the complex interdependency among RARβ-positive macrophages and stromal cells, mononuclear macrophage cell line THP-1 cells and primary HPS cells were cultured. THP-1 cells were treated with PMA (20 ng/ml) for 24 h, and then IFN (100 U/ml) with or without all-trans-RA (RA100nM) was added for 24 h to induce polarization towards an inflammatory M1 phenotype, which can activate macrophages and promote tissue damage. However, when THP-1 cells were treated with PMA (20 ng/ml) for 24 h and then treated with IL-4 (100 U/ ml) for 24 h with or without RA, the cells became polarized towards an M2 phenotype, suppressed inflammation, and contributed to wound stabilization. M1 macrophages are characterized by an IL-12^high^/IL-10^low^ phenotype, and M2 macrophages are characterized by an IL-12^low^/IL-10^high^ phenotype. Figure 5 shows that RA reduced the number of M1 macrophages secreted the inflammatory cytokines TNF-α, and IL-6 (Figure 5A and 5B), but increased the number of M2 macrophages secreted IL-10 and TGF-β (Figure 5C and 5E), as detected by ELISA of the supernatants. These data demonstrated that RA signaling promotes anti-inflammatory processes and suppresses inflammatory processes. RA reduced IL-10 expression without affecting IL-12 levels (Figure 5D), suggesting a positive effect on M2 macrophage polarization without any effect on M1 macrophage polarization.

### RA enhanced macrophage-dependent stromal cell activation

**Figure 6:**
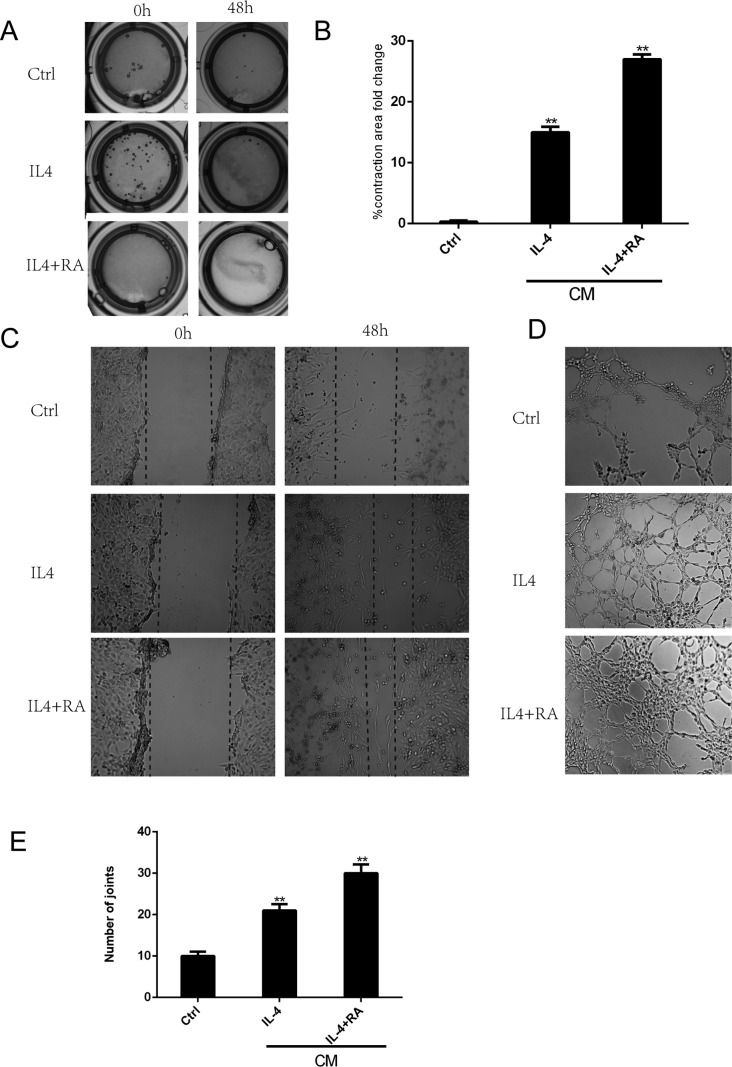
RA enhanced macrophage-dependent stromal cell activation and angiogenesis HPS cells were incubated with CM from differently treated M2 macrophages. (**A**, **B**) RA treated macrophages promoted stromal cell to contract. (**C**) RA promoted stromal cell to crawl. (**D**, **E**) RA increased tube-like structure formation in HUVECs. *p < 0.05 and **p < 0.01 as compared to the control group.

**Figure 7:**
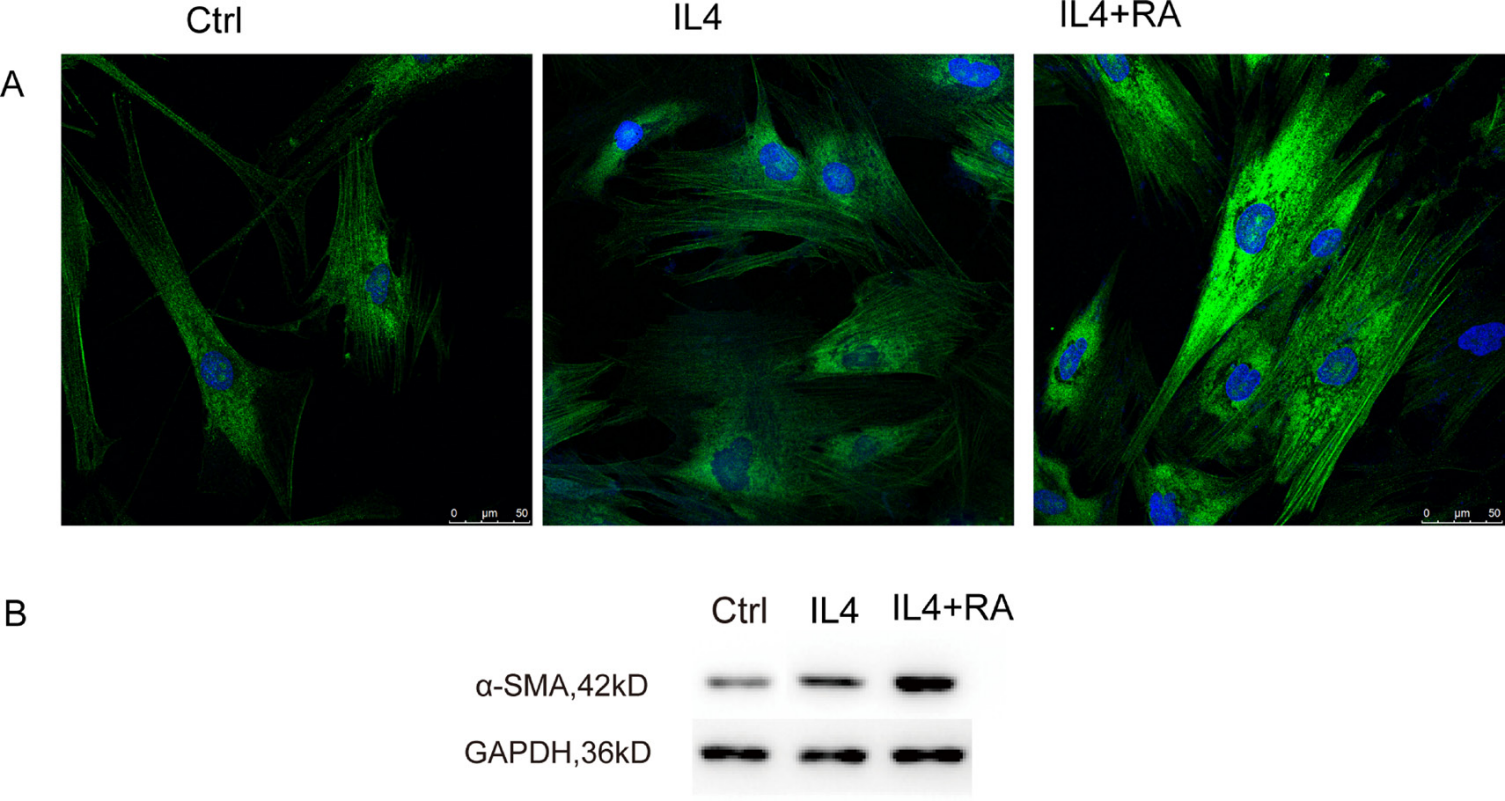
RA stimulated α-SMA organization HPS cells were incubated with CM from differently treated M2 macrophages. RA promoted α-SMA expression analyzed by immunofluorescence and western blot.

To investigate the possible role of RA in the different components of the wound-healing microenvironment, we analyzed the effects of RA-treated M2 macrophages on stromal cell activation. Primary cultured HPS cells were incubated with CM from differently treated M2 macrophages. Stromal cell activation was then analyzed by assessing alpha smooth muscle actin (α-SMA) expression (Figure 7), collagen contractility (Figure 6A and 6B), and scratch-wound assays (Figure 6C). α-SMA is a reported marker of fibroblast activation, and the quantification of collagen contractility is an established feature of activated fibroblasts [8]. Scratch-wound assays represent the migration ability of cells. RA strengthened M2 macrophage-mediated activation of stromal cells, thereby enhancing the function of the wound-healing microenvironment.

### RA promoted angiogenesis *in vitro*

M2 macrophages have been shown to increase tube-like structure formation in HUVECs. We further investigated the effect of RA-treated M2 macrophages in driving vascularization [14]. HUVECs were cultured with CM from differently treated macrophages, and their ability to form tube-like structures was examined *in vitro* (Figure 6D and 6E). We demonstrated a significant augmentation of the assembly of capillary-like structures in the cells cultured with CM from RA-treated macrophages, suggesting that RA may indirectly affect angiogenesis by altering M2 macrophage polarization.

### RARβ is required for the RA effect on Arg1 expression

**Figure 8:**
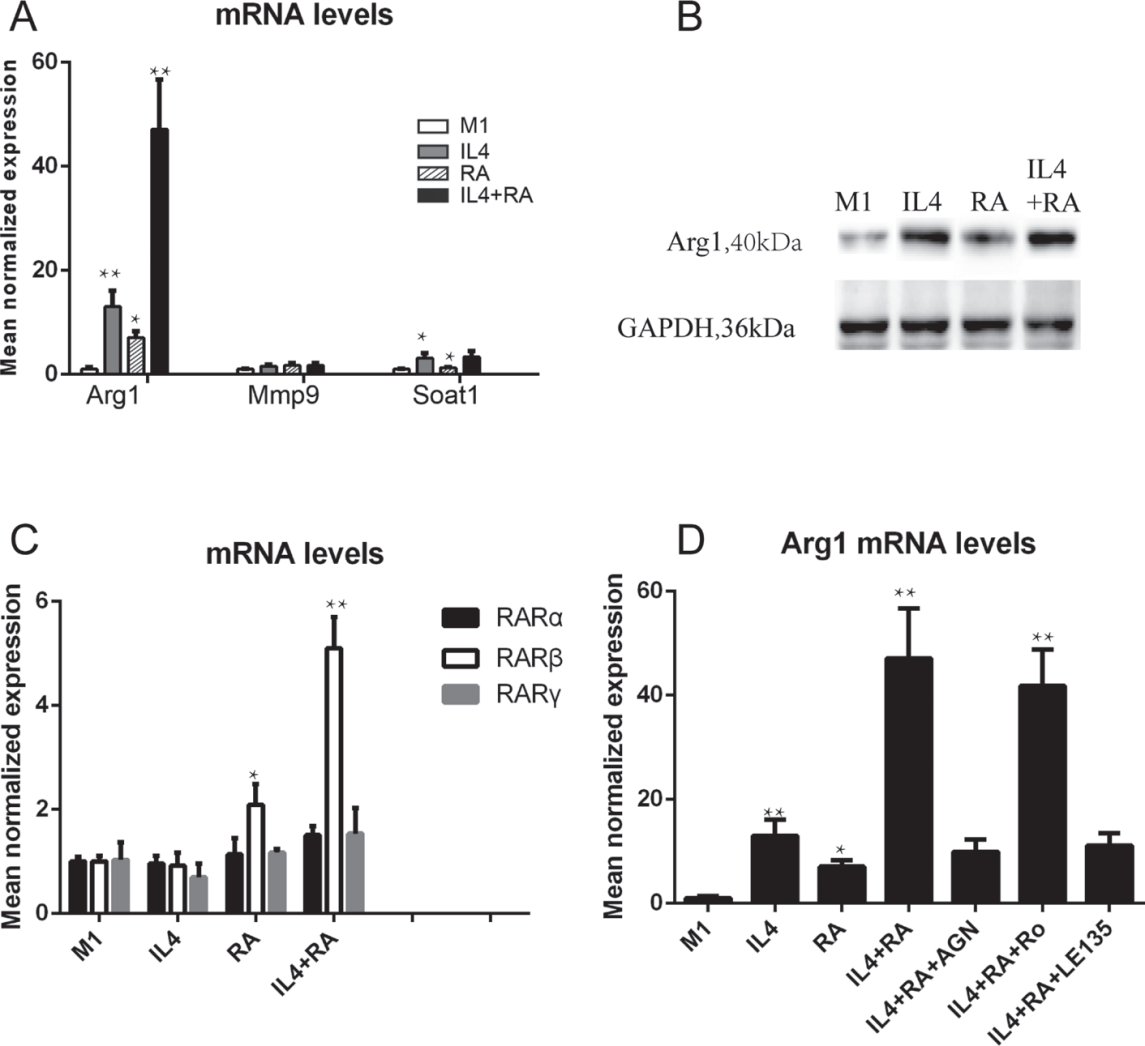
RARβ is required for the RA effect on Arg1 expression (**A**) RA promoted mRNA level of *Arg1*, but not *Mmp9* and *Soat1*. (**B**) The elevation of the Arg1 protein level in RA-treated macrophages was confirmed by western blot. (**C**) RARβ responded to RA treatment, but not RARα and γ.(**D**) An RAR pan-antagonist AGN and selectivity RARβ antagonist LE135 remarkably inhibited *Arg1* mRNA expression activated by RA, but a selective RARα antagonist Ro 41-5253 had no significant effect. *p < 0.05 and **p < 0.01 as compared to the control group.

To comprehensively investigate changes in gene expression in differently treated macrophages, *Arg1, Mmp9,* and *Soat1* mRNA expression levels were examined by real time qPCR (Figure 8A). Interestingly, the *Arg1* mRNA level was significantly elevated in RA-treated M2 macrophages, indicating that *Arg1* was a key marker for RA-enhanced macrophage activation. *Arg1* is known as a critical gene in tissue repair and has an important function in enhancing wound healing. The elevation of the Arg1 protein level in RA-treated macrophages was confirmed by western blot (Figure 8B), which suggests that a continuous supply of RA would be required for *Arg1* expression in M2-polarized macrophages. We further examined the expression of the three RAR isoforms. We found that only RARβ responded to RA treatment (Figure 8C), indicating that RARβ may be the key RAR isoform for mediating RA activation of the *Arg1* gene in macrophages. To further examine this issue, we employed pharmacological approaches and examined the RNA levels of RARs and *Arg1*. AGN is considered to be an RAR pan-antagonist, Ro 41-5253 is a selective RARα antagonist, and LE135 is an RAR antagonist with selectivity for RARβ. THP-1 macrophages were pre-treated with these antagonists to block the actions of the RAR isoforms. In Figure 8, we show that AGN and LE135 remarkably inhibited *Arg1* mRNA expression activated by RA, but Ro 41-5253 had no significant effect, suggesting the *Arg1* activation is mediated by RARβ, but not RARα and RAR γ.

## DISCUSSION

The RA signaling pathway is thought to be involved in wound healing and recovery following the inflammation process of cutaneous injury, spinal cord contusion injury, and acute kidney injury. The functions of retinoids in enhancing wound healing include epithelialization and angiogenesis. In this study, we first reported that TmLRP caused increases of RARα, RARγ, and RARβ in the wound area of the canine prostate, suggesting an involvement of RA signaling in the repair process. Our qPCR and western blotting results showed a moderate increase in the total amount of RARα and RARγ isoforms at 1, 2 and 3 weeks after surgery, but RARβ markedly increased during the first week and then decreased during the second week. RARβ expression was very low during the third week. After TmLRP, RARβs were found in macrophages, suggesting that they play a role in the inflammatory process. In the present study, double immunofluorescence staining of RARβ-positive macrophages and stromal cells confirmed the changes in macrophages, whereas the number of stromal cells gradually increased until week 3. These results suggested the possible importance of cell-to-cell interactions between macrophages and stromal cells. The mode of cell-to-cell interactions was examined *in vitro* to further study the relationships of the two types of cells with respect to healing.

After TmLRP, neutrophils quickly arrived to the wound area to break down necrotic tissue. Then, these cells were replaced by macrophages, which facilitated normal wound healing and regulated fibroblast activity. Macrophages are derived from monocytes [15], which are found in the bone marrow, blood, and spleen and can be recruited to tissue injury. Macrophages are divided into two types: inflammatory M1 macrophages and alternatively activated M2 macrophages. *In vitro*, naive macrophages can be polarized to an M1 phenotype by treatment with PMA and IFNγ; however, they can also be activated to an M2 phenotype by treatment with PMA and IL4. M1 macrophages are recruited to the prostate wound area and amplify the inflammatory responses. Then, these macrophages are replaced by M2 macrophages that promote repair [9]. M2 macrophages contribute to suppressing inflammation through secretion of IL-10 and contribute to wound stabilization by their role in matrix-remodeling. Using THP-1 cells, we found that RA reduced the secretion of TNF-α and IL-6 by M1 macrophages but increased the secretion of IL-12 by M2 macrophages. We demonstrated that RA regulated macrophage activation by suppressing inflammatory M1 macrophages and stimulating M2 macrophages via RA signaling in the prostate wound-healing process.

Following the inflammatory phase, stromal cells containing fibroblasts migrated into the wound and the activation of these cells was of particular importance for healing of the prostate. Angiogenesis also occurred at this point, which is essential to wound closure. Our current study demonstrated that CM from RA-treated M2 macrophages promoted stromal cell migration and activation (Figures 6 and 7). Angiogenesis was also promoted *in vitro* by the CM (Figure 6). Arg1, a gene critical to wound healing, was remarkably increased in the RA-treated macrophages. RARβ was demonstrated to be the key isoform.

In conclusion, three Isoforms of RAR were increased after TmLRP in the canine prostate. RARβ was expressed in the macrophages, enhanced the function of anti-inflammatory macrophages and promoted stromal cell activation and angiogenesis. These results indicated that RA signaling caused macrophages to promote stromal cell activation and angiogenesis and enhanced the wound-healing process after TmLRP. RARβ was the key isoform in this process. In this study, we also found that RARγ was present in the basal cells of prostate epithelium, and further studies on RARγ, basal cells, and their relationship to re-epithelialization are required. The role of the RA signaling pathway in the wound-healing process requires a more comprehensive understanding. This knowledge may offer new methods to accelerate the wound-healing process and reduce complications. If we apply the RA to the catheter, it may improve the prostate wound repair.

## MATERIALS AND METHODS

### Animal experiments

Twelve male adult beagle dogs, aged 2 years and weighing 13-17 kg, were used. During the operation, the canines were generally anesthetized with 3% sodium pentobarbital (1 ml/kg) and placed in a supine position on the operating table. A 3-cm lower abdominal midline incision was made to expose the bladder. The anterior wall of the bladder was incised and placed onto a 26F continuous-flow resectoscope with a Tm: YAG laser system. Under saline irrigation, thulium laser resection of the prostate was performed as described previously [3, 11]; then the bladder and the abdominal wall were closed. No transurethral catheter was required. One, two, and three weeks after the operation, the canines were killed, and unoperated canines served as controls. Prostates were removed and divided into two halves, one was stored in 10% buffered formalin, and the other half was immediately placed in liquid nitrogen.

### Cell culture

Normal human prostate stromal (HPS) cells were cultured from human prostatic stromal tissue as described earlier [12]. Passage 3–5 cells were used in all subsequent experiments. The THP-1 cell line was obtained from American Type Culture Collection (ATCC, VA, USA). Human Umbilical Vein Endothelial Cells (HUVEC) were a generous gift from Prof. Ju Zhang (Nankai University, Tianjin, China). The THP-1 and HUVEC cell lines were maintained in RPMI-1640 medium (HyClone, UT, USA) supplemented with 10% fetal bovine serum (Gibco, CA, USA), 100 U/ml penicillin, and 100 mg/ml streptomycin (Gibco, CA, USA). HPS cells were maintained in DMEM/high glucose medium (HyClone, UT, USA) supplemented with 20% fetal bovine serum (Gibco, CA, USA), 100 U/ml penicillin, and 100 mg/ml streptomycin (Gibco, CA, USA). All cell lines were cultured at 37°C in a 5% CO2 environment.

### Preparation of conditioned media

Conditioned media (CM) were obtained from differently activated THP-1. Cells were serum starved and incubated for 48 h before collection of the CM. supernatant were harvested as CM, clarified by centrifugation, and used freshly

### Western blotting

For protein analysis, tissue was removed from the wound and the nearby area of the prostate after TmLRP, cut into small pieces, and then lysed using cell lysis buffer (Beyotime, China). The cells were washed with cold PBS twice and solubilized in lysis buffer. The lysates were centrifuged at 13,000 *g* at 4°C for 5 min. Proteins were separated with discontinuous SDS-PAGE and transferred onto a nitrocellulose membrane (Mini TransBlot electrophoretic transfer cell; Bio-Rad, CA, USA). The membranes were blocked at room temperature for 1 h with blocking solution. The membranes were then incubated overnight at 4°C with the following primary antibodies: RARα (1:1000 dilution; rabbit polyclonal antibody; Santa Cruz, CA, USA), RARβ (1:1000 dilution; rabbit polyclonal antibody; nuclear staining; Santa Cruz, USA), RARγ (1:1000 dilution; rabbit monoclonal antibody; Cell Signal), α-SMA (1:1000 dilution; mouse monoclonal antibody; Sigma-Aldrich, USA), and GAPDH (1:1000 dilution; mouse monoclonal antibody; Beyotime, China) as a control. After two 10-min washes in TBST, the membranes were incubated for 1 h at room temperature with a peroxidase-conjugated anti-rabbit or anti-mouse secondary antibody at a 1: 10,000 dilution (Jackson ImmunoResearch Laboratories, USA) in blocking solution. Detection was performed by enhanced chemiluminescence (ECL) using a Western Blotting Luminol Reagent (Beyotime, China) according to the manufacturer's instructions. The bands were then quantified by scanning densitometry (Tanon Image System, Tanon, China).

### Immunohistochemistry

Canine prostate tissues were fixed in 10% (v/v) formaldehyde, embedded in paraffin, and cut into 5-μm tissue sections. Prostate sections were deparaffinized in xylene solution and rehydrated using gradient ethanol concentrations. Immunostaining was performed as described previously [13]. The sections were incubated with the following primary antibodies overnight at 4°C: RARα (1:300 dilution; rabbit polyclonal antibody; nuclear staining; Santa Cruz, CA, USA), RARβ (1:300 dilution; rabbit polyclonal antibody; nuclear staining; Santa Cruz, USA), RARγ (1:100 dilution; rabbit monoclonal antibody; nuclear staining; Cell Signal, USA), and vimentin (1:200 dilution; rabbit monoclonal antibody; cytoplasmic staining; Abcam, UK). Following a thorough rinse, the sections were incubated with secondary antibodies (peroxidase-conjugated anti-rabbit or anti-mouse secondary antibody at 1:1000(Jackson ImmuoResearch Laboratories, USA)) for 60 min at room temperature. Nuclei were counterstained with hematoxylin. Finally, the tissue slides were dehydrated in bath solutions. Negative controls for these immunohistochemical procedures were incubated with nonimmune serum instead of the primary antibodies, which resulted in no detectable staining.

### Immunofluorescence

To measure immunofluorescence in tissues, prostates sections were fixed in 4% paraformaldehyde (PFA) solution at 4°C for 1 h, and washed with PBS containing 0.05% Tween 20, then these slides were blocked with 5% bovine serum albumin for 2 h at room temperature. The prepared slides were stained with primary antibodies overnight at 4°C. The primary antibodies included RARβ (1:300 dilution; rabbit polyclonal antibody; nuclear staining; Santa Cruz, USA), CD68(1:100 dilution, mouse monoclonal antibody; cytoplasmic staining; Abcam, Cambridge, UK)and vimentin (1:1000 dilution; mouse monoclonal antibody; cytoplasmic staining; Sigma-Aldrich, USA). After washing, the slides were then stained with secondary antibody (Alexa Fluor 488-labeled Goat Anti-Mouse IgG and Alexa Fluor 555-labeled Donkey Anti-Rabbit IgG, Beyotime Biotechnology, China). Nuclei were counterstained with 40,6-diamidino-2-phenylindole dihydrochloride hydrate (DAPI; Beyotime Biotechnology, China). To measure immunofluorescence in cells, HPS cells were cultured on a glass slide in a 24-well plate and incubated with different conditioned media (CM). According to the manufacturer's instructions, HPS cells were washed with PBS and then fixed with 4% ice-cold PFA for 15 min. A mouse monoclonal antibody to human α-SMA (1:500, Sigma-Aldrich, USA)) was applied overnight at 4°C followed by a 1-h incubation with Alexa Fluor 488-labeled goat anti-mouse IgG (1:1000). The slides were observed under a fluorescence microscope.

### Real-time quantitative polymerase chain reaction (qPCR)

Total RNA from prostate wound specimens or cell lines was extracted using the TRIzol reagent (Life Technologies). Reverse transcription PCR was performed using PrimeScript RT Master Mix (Takara, Japan) according to the manufacturer's instructions. Real-time qPCR was performed using SYBR Premix Ex Taq (Takara, Japan). GAPDH was used as an internal control. qPCR conditions included pre-denaturation at 95°C for 30 s, followed by 40 cycles of denaturation at 95°C for 5 s and annealing at 60°C for 30 s. The expression fold-change was calculated using the 2ΔΔCt method.

### Elisa

Cytokine production in macrophage supernatants was measured by commercially available ELISA Kits (TNF-α, TGF-β1, IL-6, IL-10, and IL-12p40) according to the manufacturer's instructions (RayBiotech, USA).

### Tube-like formation assay

All experiments were performed using growth factor-reduced Matrigel at a concentration of 1 mg/ml. First, 50 μl of Matrigel was added to each well of a 96-well plate and then placed in a humidified incubator at 37°C for 30 min. HUVECs (2 × 10^4^ cells/well) were added to the Matrigel-coated plates at a final volume of 200 μl. The effects on the morphogenesis of endothelial cells were recorded after 6 h with an inverted microscope equipped with CCD optics and a digital analysis system. The results were quantified by counting the joint in several fields.

### Collagen gel contraction assay

For this assay, 1.5 × 10^5^ prostatic stromal cells (HPS) were embedded in 500 μl of DMEM solution containing 1 mg/ml collagen and placed into the wells of 24-well plate (Corning, Costar). Cells containing gel were allowed to polymerize at 37°C for 1 h before the gel disc was detached from the wall using a sterile tip. Subsequently, 1 ml of serum-free medium or CM was added to the wells. The floating gels were incubated at 37°C with 5% CO2 for 48 h, and the gel area was then quantified. To determine the degree of collagen gel contraction, images of the gels were obtained, and the estimated area of each gel (number of pixels) was analyzed with Adobe Photoshop.

### Scratch-wound assays in stromal cells

We seeded HPS cells in six-well plates. Cell growth was allowed to continue until confluence was reached. The cell monolayer was then scratched with a 10-ml pipette tip, and dislodged cells were washed away with PBS. The cells were incubated in serum-free DMEM/high glucose medium, and images were acquired every 24 h. The distance of cell gaps was measured from each side of the scratch at three spots within the same scratch.

### Statistical analysis

All experiments were performed multiple times with at least 3 sets of experiments that revealed similar results. All data are presented as means ± standard deviations (SD). A non-parametric test was used to analyze the data with the statistical software SAS 6.0 (SAS Institute Inc., USA). *P* values < 0.05 were considered statistically significant.
